# Chimeric antigen receptor T cell therapy based on stem cell‐like memory T cells enhances anti‐tumour effects in multiple myeloma

**DOI:** 10.1002/ctm2.70264

**Published:** 2025-03-05

**Authors:** Zhaoyun Liu, Xintong Xu, Yihao Wang, Panpan Cao, Jia Song, Kai Ding, Hui Liu, Rong Fu

**Affiliations:** ^1^ Department of Hematology Tianjin Medical University General Hospital Tianjin China; ^2^ Tianjin Key Laboratory of Bone Marrow Failure and Malignant Hemopoietic Clone Control Tianjin China; ^3^ Haihe Laboratory of Cell Ecosystem Tianjin China; ^4^ Tianjin Institute of Hematology Tianjin China; ^5^ State Key Laboratory of Experimental Hematology Tianjin China

1

To the Editor,

Multiple myeloma (MM) is the second largest malignant tumour of the haematological system.^1^ Nowadays, chimeric antigen receptor T‐cell (CAR‐T) therapy has become a revolutionary approach to the treatment of MM, significantly prolonging progression‐free survival (PFS) and overall survival (OS) of MM patients.^2^, ^3^ However, ultimately, most patients inevitably face the outcome of Relapse and drug resistance. Stem cell‐like memory T cell (T_SCM_) cells are a population of long‐lived memory T cells with the capacity for self‐renewal and differentiation.^4^, ^5^, ^6^ However, T_SCM_ cells are extremely low in proportion, and current technology still does not allow for easy access to sufficient quantities of T_SCM_ cells.^7^ Currently, the therapeutic strategy of CAR‐T_SCM_ has been actively pursued in solid tumours, but it is still rare in MM.

We first investigated the quantity and function of bone marrow T_SCM_ cells in MM patients with flow cytometry (FCM). We chose ‘CD3^+^T_SCM_ events/CD3^+^T events’ and ‘CD8^+^T_SCM_ events/CD8^+^T events’ to show the percentage of T_SCM_ and to ensure the comparability of the statistics results. The results revealed that the percentage of CD3^+^T_SCM_ cells was significantly reduced in the newly diagnosed MM (NDMM) compared to both the health control (HC) (0.51 ± 0.31% vs. 0.98 ± 0.39%) and the complete response (CR) (0.51 ± 0.31% vs. 0.92 ± 0.54%) (*p *<  .0001, *p *= .0005). The percentage of CD3^+^CD8^+^T_SCM_ cells, which was reduced in the NDMM compared to both the HC (0.41 ± 0.28% vs. 0.97 ± 0.40%) and the CR (0.41 ± 0.28% vs. 0.61 ± 0.36%) (*p *<  .0001, *p *= .0132) (Figure S1A,B).

Then, we found that the expression levels of Perforin and Granzyme B were significantly higher in CD3^+^T_SCM_ cells compared to CD3^+^T cells (42.26 ± 3.69% vs. 15.32 ± 2.75%, *p *<  .0001; 57.18 ± 6.38% vs. 21.56 ± 3.38%, *p *<  .0001); similarly, higher in CD3^+^CD8^+^T_SCM_ cells than in CD3^+^CD8^+^T cells (47.77 ± 3.18% vs. 18.37 ± 3.89%, *p *<  .0001; 50.30 ± 4.12% vs. 28.84 ± 3.49%, *p *<  .0001) (Figure S1C).

Afterwards, we took the bone marrow from MM patients who were candidates for B‐cell maturation antigen (BCMA)‐CART treatment. After aspiration, part of the bone marrow from these patients was used for their own clinical treatments and the rest was used in this experiment. Then we induced the expansion of T_SCM_ cells in vitro and FCM was used to detect the number and function of T_SCM_ cells (Figure 1A).

**FIGURE 1:**
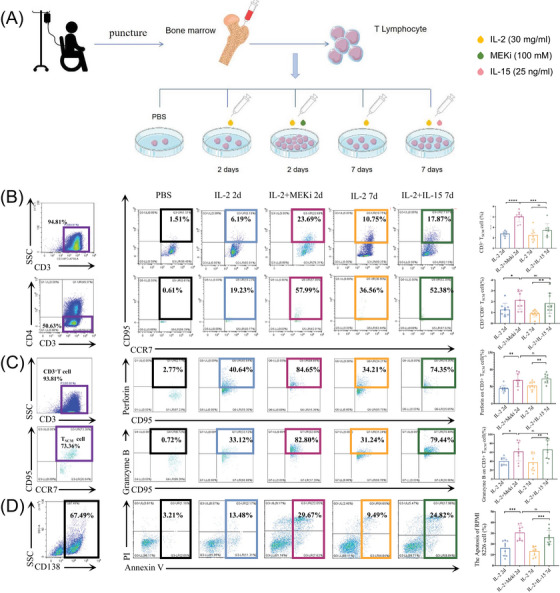
By combining interleukin (IL)‐2 and MEKi in culture for 2 days, T_SCM_ cells could be effectively induced and expanded in vitro and exerted potent anti‐tumour effects. (A) Schematic diagram of the amplification system for constructing T_SCM_ cells in vitro. (B) Flow cytometry (FCM) assay for comparison of the quantity of CD3^+^T_SCM_ cells and CD3^+^CD8^+^T_SCM_ cells in four in vitro induced expansion T_SCM_ cells modalities. (C) FCM assay for comparison of the function of CD3^+^T_SCM_ cells and CD3^+^CD8^+^T_SCM_ cells in four in vitro induced expansion T_SCM_ cells modalities. (D) T_SCM_ cells induced and expanded by different methods were co‐cultured with multiple myeloma (MM) cell line RPMI‐8226, and flow cytometry was applied to detect the apoptosis rate of MM cell line RPMI‐8226 cells after 48h. *p <  .05; **p <  .01; ***p <  .001; ****p <  .0001; MEKi, MEK1/2 inhibition; ns, not significant.

It has been demonstrated that MEK1/2 inhibition (MEKi) can be used to perform T_SCM_ cell expansion and can inhibit CAR‐T depletion and differentiation.^8^, ^9^ We found that the percentages of CD3^+^T_SCM_ cells were significantly higher (6.04 ± 1.02%, *P *<  . 05) after 2d of induction by interleukin (IL)‐2 and MEKi (Figure 1B).

As for the function, the expression levels of Perforin and Granzyme B in T_SCM_ cells were significantly increased in IL‐2 and MEKi group (68.92 ± 19.20%, 61.60 ± 21.01%, *p *<  .05) (Figure 1C). What's more, when co‐cultured with MM cell line RPMI‐8226 after 48 h, the anti‐tumour ability of T_SCM_ cells induced and expanded by IL‐2 and MEKi was significantly enhanced when compared with other groups (Figure 1D).

Then, D0 (the day the CAR‐T_SCM_ cells were constructed) was selected for subsequent experiments to activate T cells with T Cell TransAct™. By co‐culturing with the RPMI8226 cell line in the ratio of effector cells to target cells of 1:1 for 5h and 24h, we found that virus transfection can significantly improve the tumour‐killing ability (Figure S2).

Finally, we validated it with mouse models. The RPMI‐8226 Luciferase Overexpression stable transcript strain was first constructed(Figure S3), and 16 4–6‐week‐old NSG mice were selected and injected (3×10^6^ cell/mouse) (Figure 2A). The experimental groups were designed as Group 1 (G1, PBS control), G2 (BCMA‐CART), G3 (CAR‐T_SCM_) and G4 (T_SCM_ cell), with the treatment dose of 5×10^6^/mouse. The tumour load of mice in each group were observed and calculated by in vivo imaging at D0, D4, D8, D14, D33 and D42 (Figure 2B,C ), and blood was taken from the tail vein to assess the expression of CAR and the liver and kidney index of mice at D33 among the groups. We found a significant reduction in tumour load when respectively compared G2、G3 and G4 to G1 on D42 (G1 vs. G2:4.41×10^9^, 95% confidence interval [95%CI] 3.62–5.22×10^9^, *p *<  .0001; G1 vs. G3:4.38×10^9^, 95%CI 3.61–5.17×10^9^, *p *<  .0001; G1 vs. G4:4.09×10^9^, 95%CI 3.28–4.91×10^9^, *p *<  .0001). Among them, the tumour load reduction was more significant in G2 and G3, but no significant difference was demonstrated between these two groups (*p *> .05) (Figure 2D). At this time, no difference was seen in CAR expression between G2 and G3 (*p *> .05) (Figure 2E).

**FIGURE 2:**
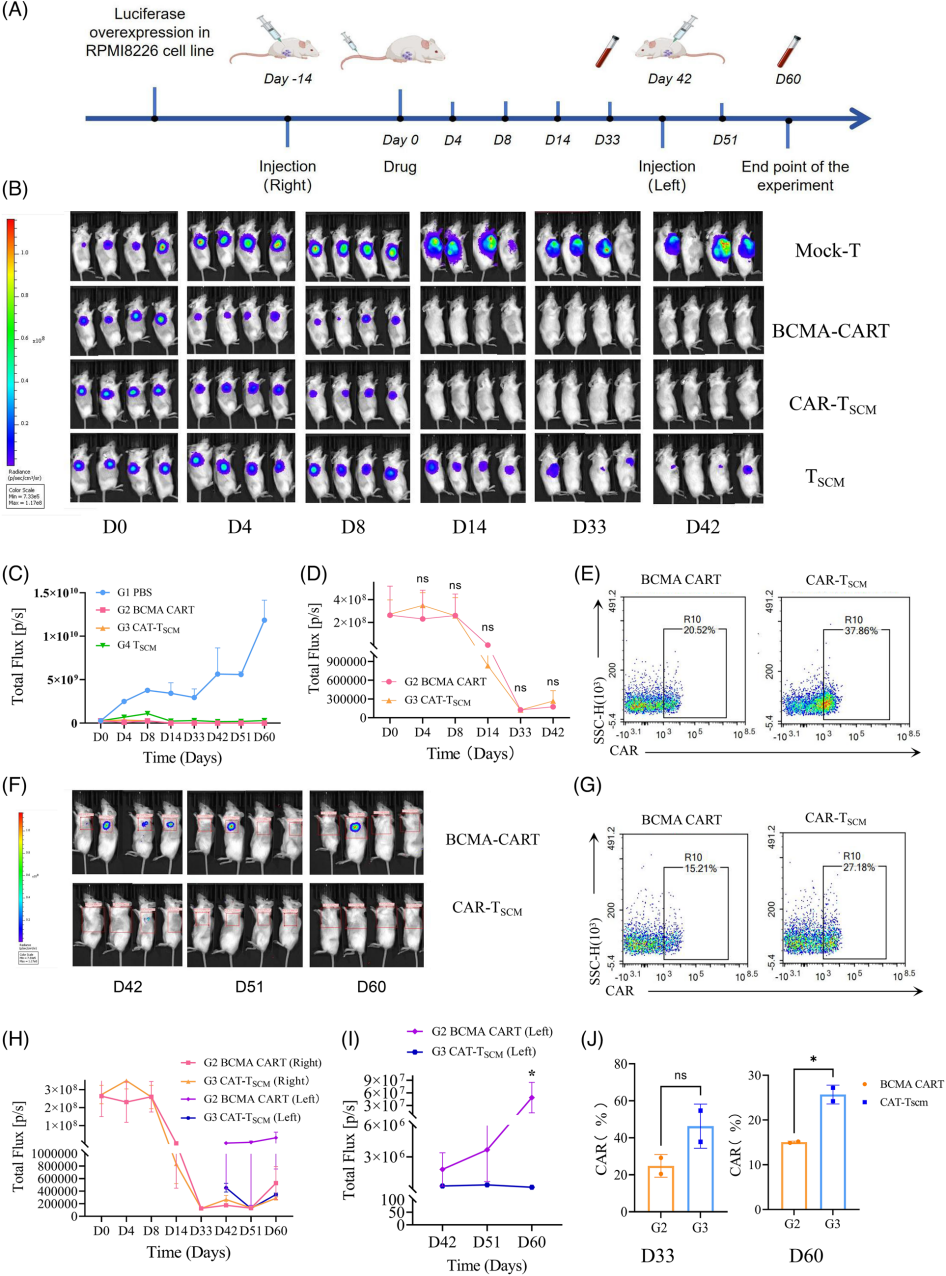
(A) Timeline of animal experiments. (B) In vivo imaging was performed at D0, D4, D8, D14, D33 and D42 after injection of PBS, BCMA‐CART cell, CAR‐T_SCM_ cell and T_SCM_ cell, respectively. (C) Tumour load was expressed in terms of fluorescence intensity, and the differences in tumour load were compared among the four groups of mice. (D) To compare the difference in tumour load at D0, D4, D8, D14, D33 and D42 between G2 and G3. (E) CAR expression was determined by flow cytometry (FCM) in G2 and G3 mice at D33. (F) Live imaging of G2 and G3 at D42, D49 and D60 after contralateral subcutaneous tumour formation. (G) CAR expression was determined by FCM in G2 and G3 at D60. (H) Comparison of tumour load in bilateral tumour mounds of mice in G2 and G3 throughout the experiment. (I) Comparison of tumour loads of D42, D49, and D60 on the contralateral side of mice in G2 and G3. (J) Analysis of CAR expression in G2 and G3 at D33 and D60. Note: G1 (PBS control group), G2 (BCMA‐CART treatment group), G3 (CAR‐T_SCM_ treatment group) and G4 (T_SCM_ cell treatment group); *p <  .05; ns: not significant.

After that, contralateral subcutaneous tumour formation was performed in the G2 and G3 on D42, and the tumour load were observed in D42, D51, and D60(Figure 2F,H). We noticed that the tumour load seen in the G3 was significantly reduced compared to the G2 (4.88×10^7^, 95%CI 0.09–9.67×10^7^, *p *= .045) (Figure 2I). This suggested a better long‐term therapeutic efficacy of the G3. At this time, the expression of CAR in the G3 was higher than that in the G2 on D60 (Figure 2G,J). Combining the results of the two blood tests, we found that the G3 could exert a stronger and longer‐lasting anti‐tumour effect and does not affect liver or kidney index in mice (Figure S4).

## 2 CONCLUSION

At present, the research progress of CAR‐T_SCM_ is still mainly limited to solid tumours, such as lung cancer, hepatocellular carcinoma, ovarian cancer, etc.^10^ Therefore, we focused on MM, expanded a higher proportion of T_SCM_ cells and produced CAR‐T_SCM_ cells, and found through ex vivo and in vivo experiments that it may be more durable than traditional CAR‐T, which may have exerted a more durable tumour killing effect, perhaps enabling MM mice to obtain deep disease remission and providing new ideas for clinical treatment.

## AUTHOR CONTRIBUTIONS

Zhaoyun Liu, Xintong Xu and Yihao Wang performed experiments, analyzed and interpreted data and wrote the manuscript. Panpan Cao, Jia Song, Kai Ding and Hui Liu analyzed and interpreted data, and revised the manuscript. Rong Fu designed the study, interpreted data and wrote the manuscript. All authors reviewed the manuscript.

## CONFLICT OF INTEREST STATEMENT

The authors declare no conflict of interest.

## FUNDING INFORMATION

This work was supported by the Key research and development projects of the Ministry of Science and Technology (grant no. 2024YFC2510500), the National Natural Science Foundation Project (grant nos. 82270142 and 81900131), the Tianjin Municipal Natural Science Foundation (grant no. 24ZGSSSS00050), the Tianjin Science and Technology Planning Project (grant nos. 24ZXGZSY00090 and 24ZXGQSY00020), Tianjin Municipal Health Commission Project (grant no. TJWJ2023XK003) and China Postdoctoral Science Foundation (grant no. 2023M742624).

## ETHICS STATEMENT

Our study protocol was reviewed and approved by the Ethics Committee of the General Hospital of Tianjin Medical University in accordance with the guidelines of the Declaration of Helsinki of the World Medical Association, and all subjects voluntarily signed an informed consent form (Ethics No.IRB2023‐KY‐294). All mouse experiments were reviewed and approved by the Ethics Committee of Tianjin Medical University General Hospital in accordance with the guidelines of the Declaration of Helsinki of the World Medical Association (Ethics No.IRB2023‐DWFL‐363).

## Supporting information



Supporting Information

Supporting Information

Supporting Information

Supporting Information

Supporting Information

## Data Availability

Any additional information required to reanalyze the data reported in this paper is available from the corresponding author upon reasonable request.
