# Supplementary figures and images for: Chimeric antigen receptor T cell therapy based on stem cell‐like memory T cells enhances anti‐tumour effects in multiple myeloma
Source: Clin Transl Med. 2025 Mar 5;15(3):e70264. doi: 10.1002/ctm2.70264 (PMC11882385; doi:10.1002/ctm2.70264)

Figure S1

a

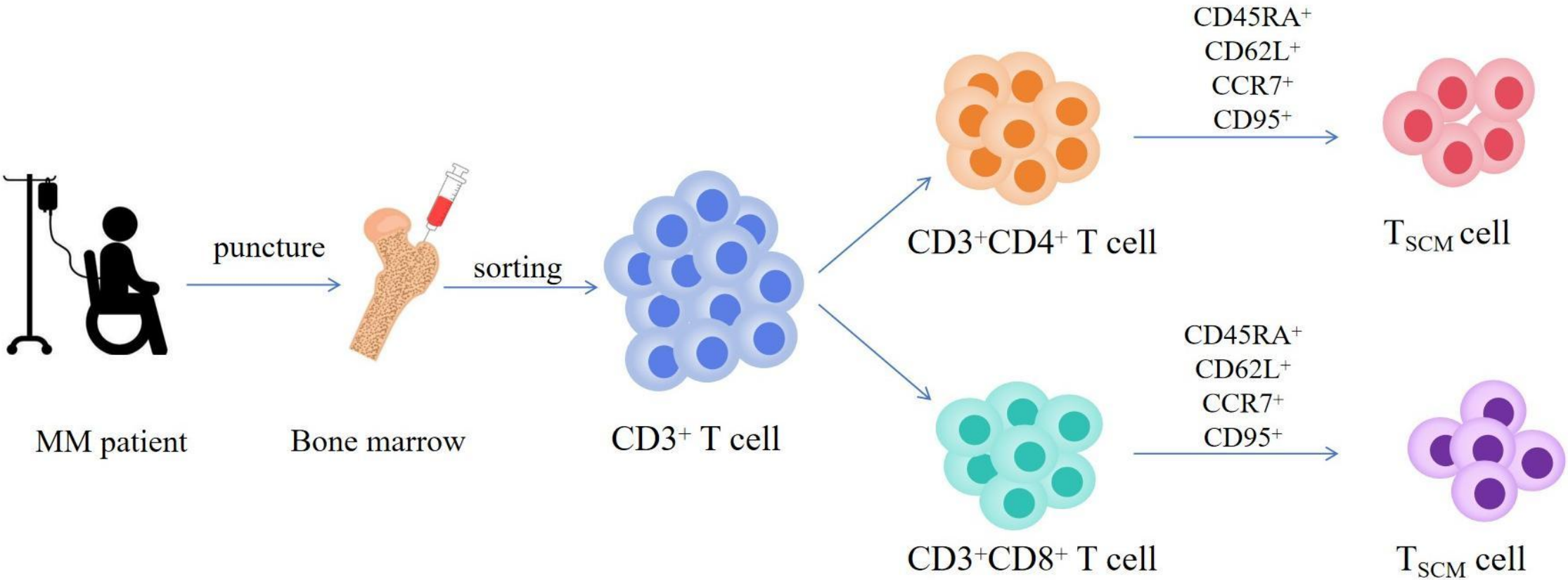

b

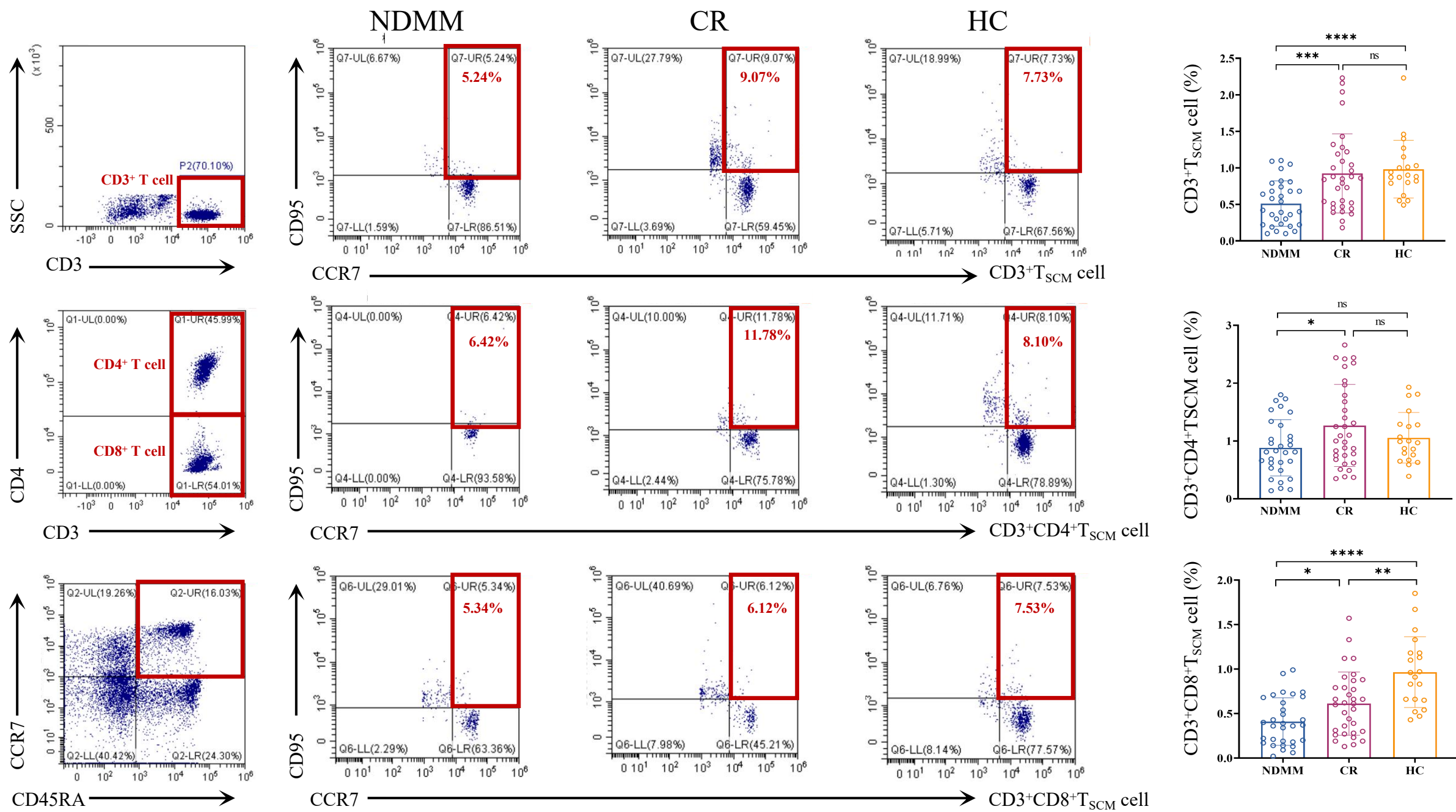

c

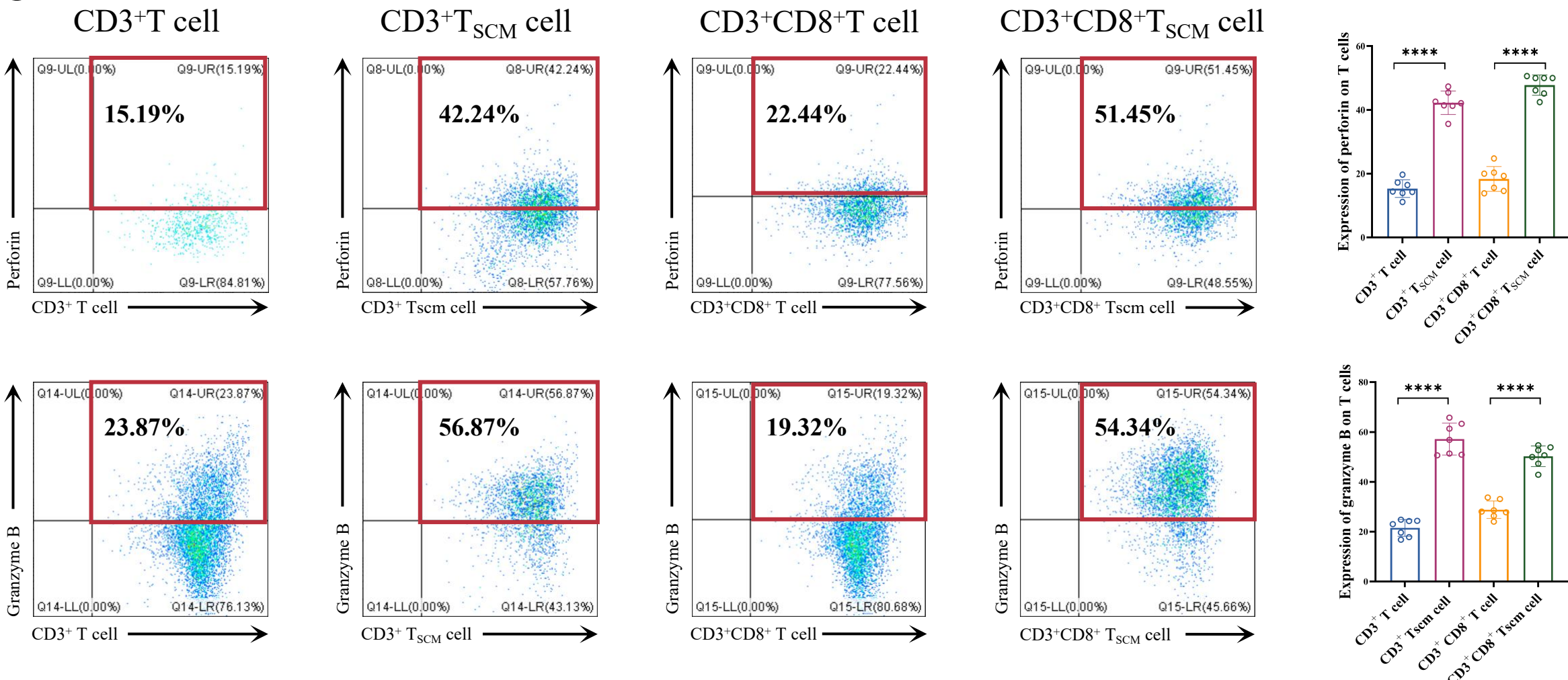

Supplement: Supplementary file 1 — Supporting Information [file CTM2-15-e70264-s005.pdf]

# Figure S2

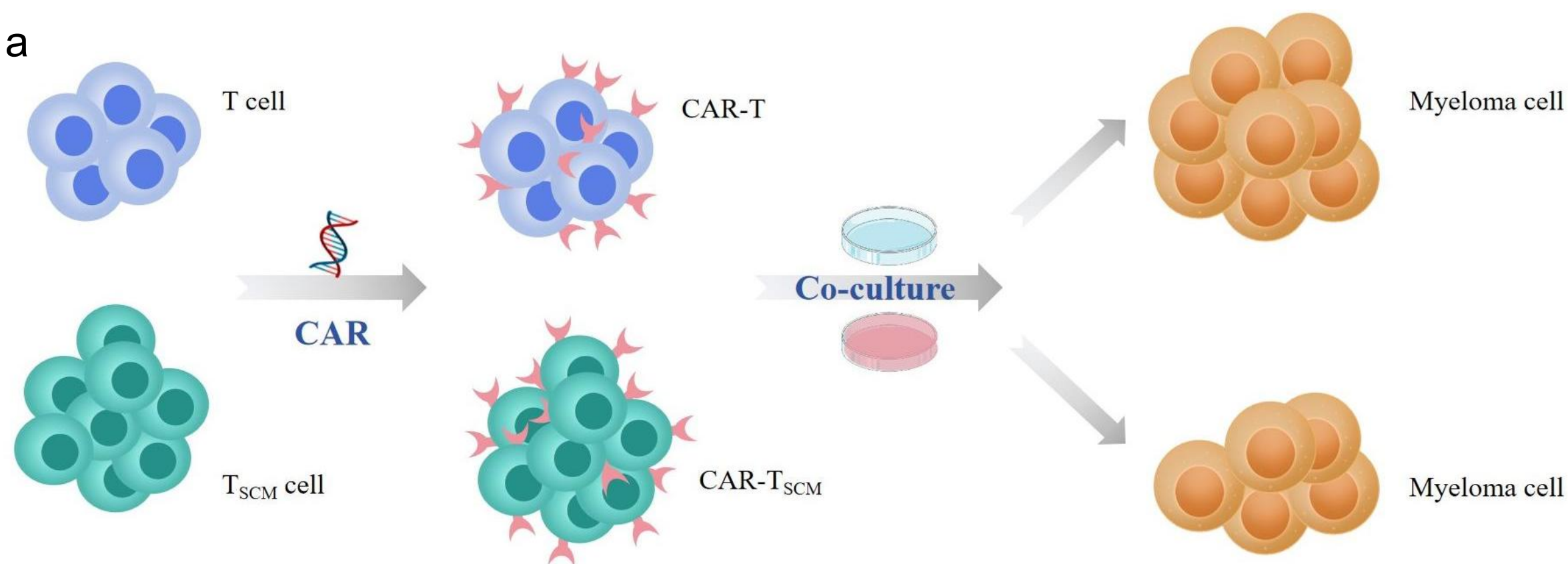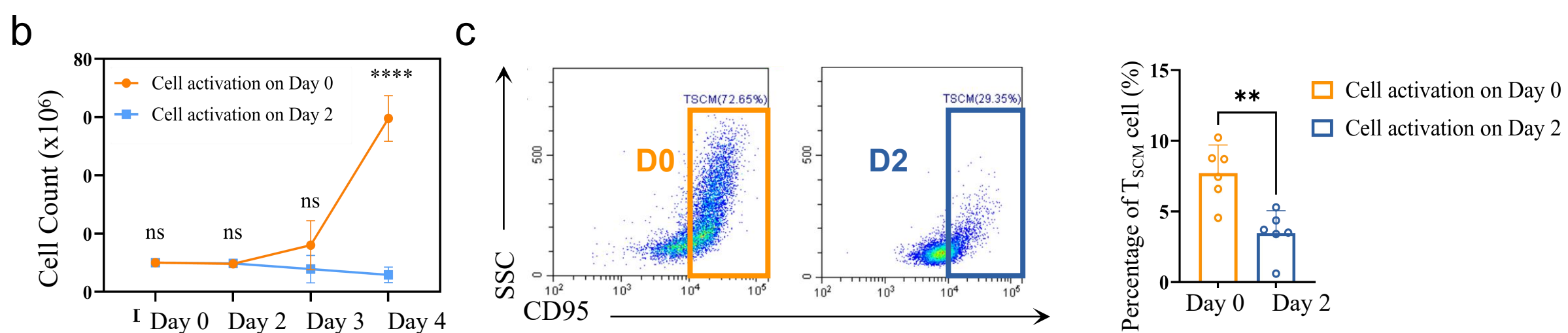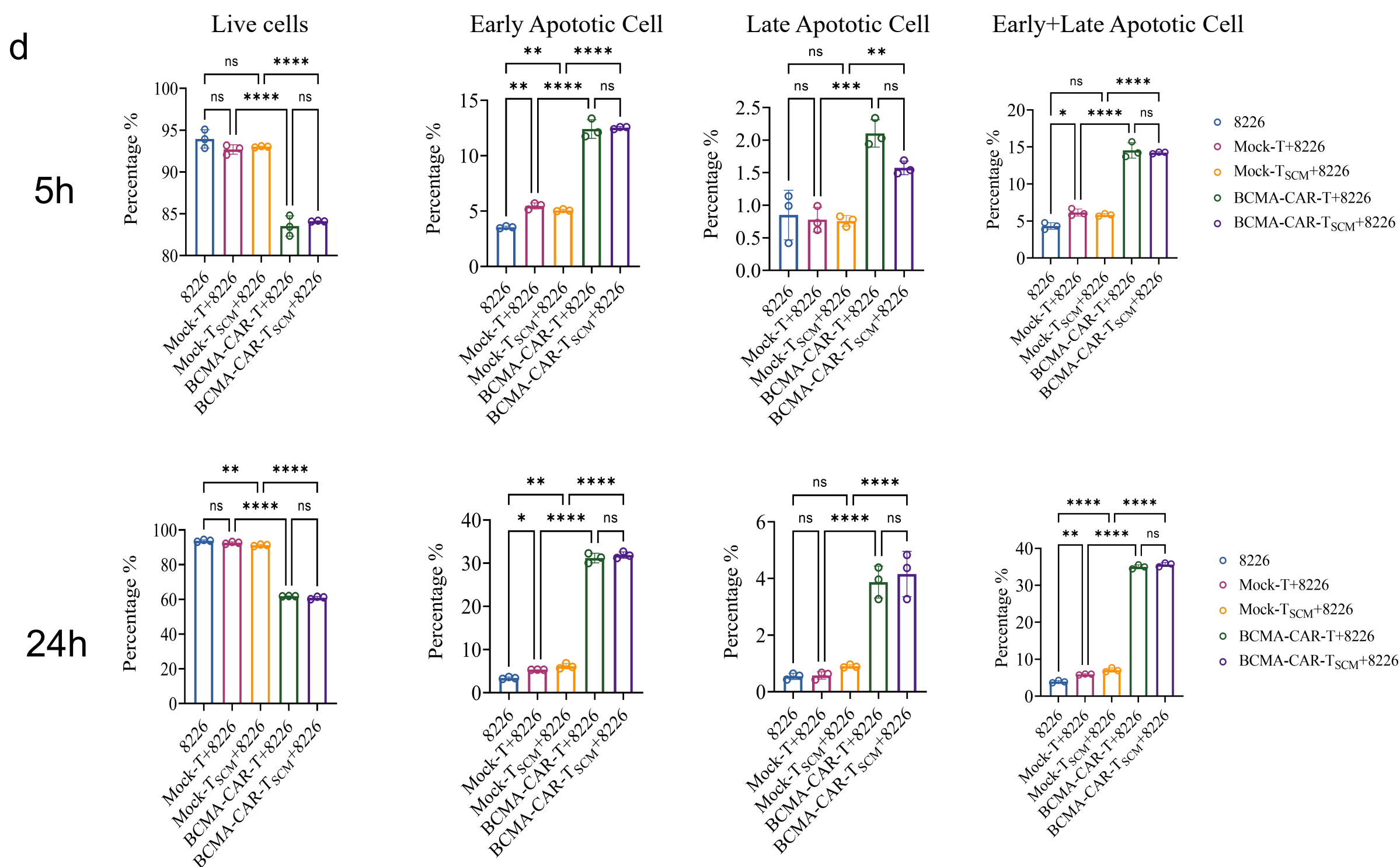

Supplement: Supplementary file 2 — Supporting Information [file CTM2-15-e70264-s002.pdf]

# Figure S3

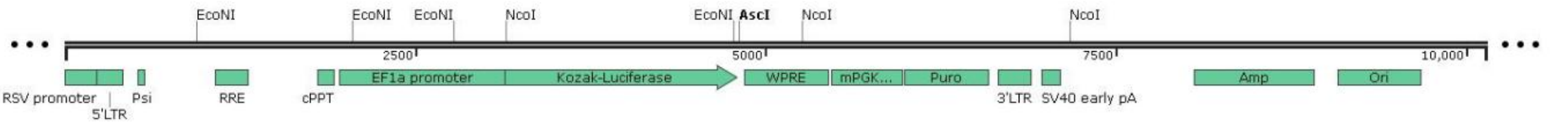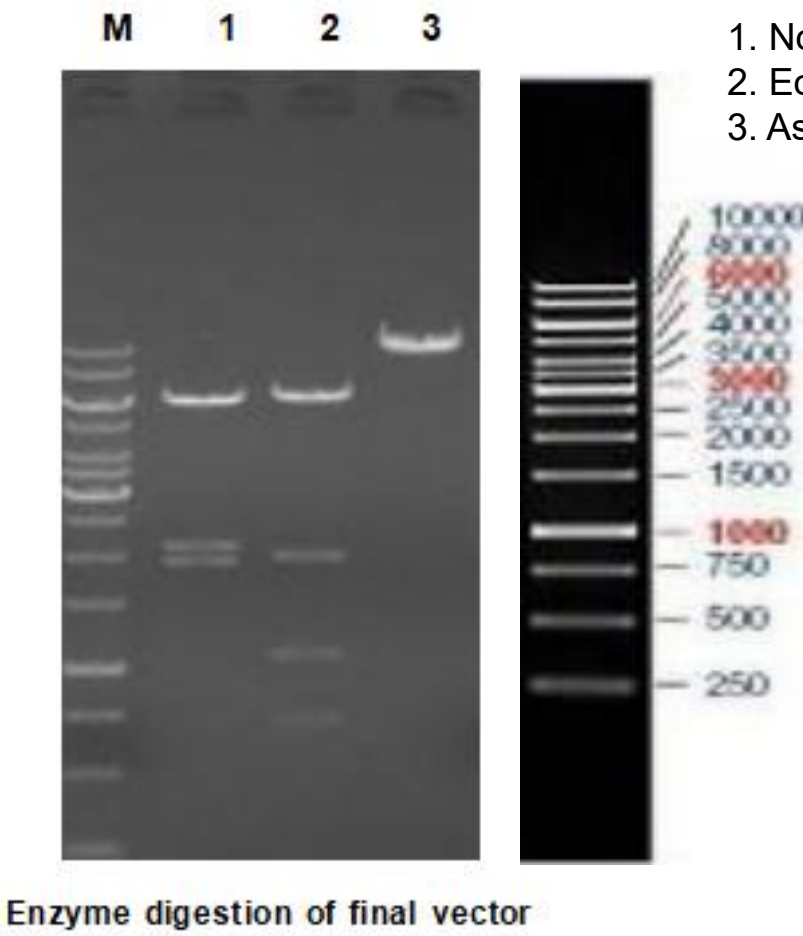

- 1. NcoI: 6.1/2.1/ 1.9
- 2. EcoNI: 6.3/2.0/ 1.1/0.7
- 3. Ascl: 10.1

## Luciferase overexpression-100x

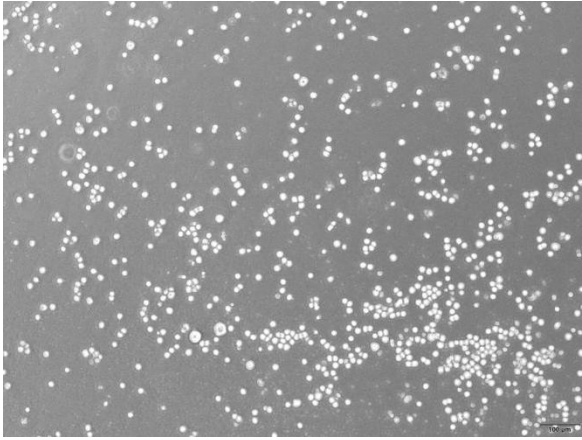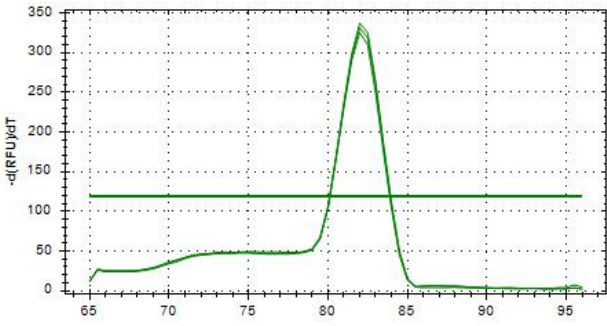

Luciferase

## Control-100x

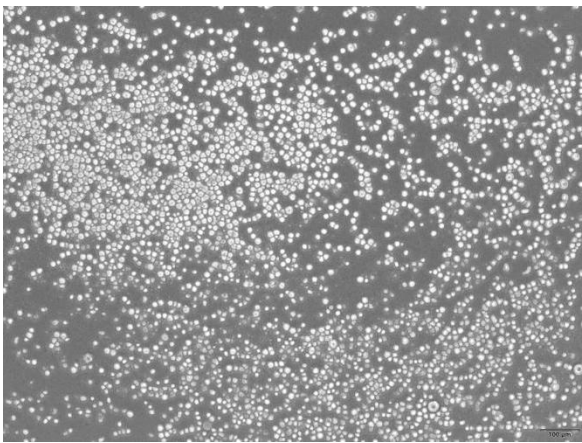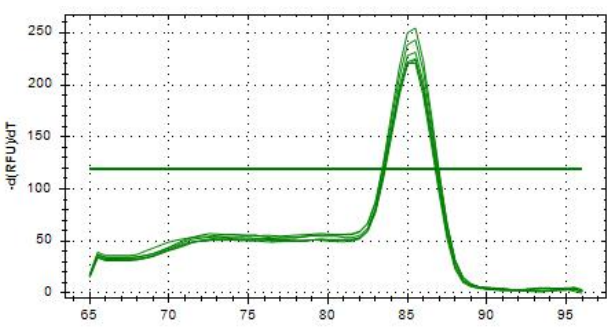

hGAPDH

Supplement: Supplementary file 3 — Supporting Information [file CTM2-15-e70264-s003.pdf]

Figure S4

D33

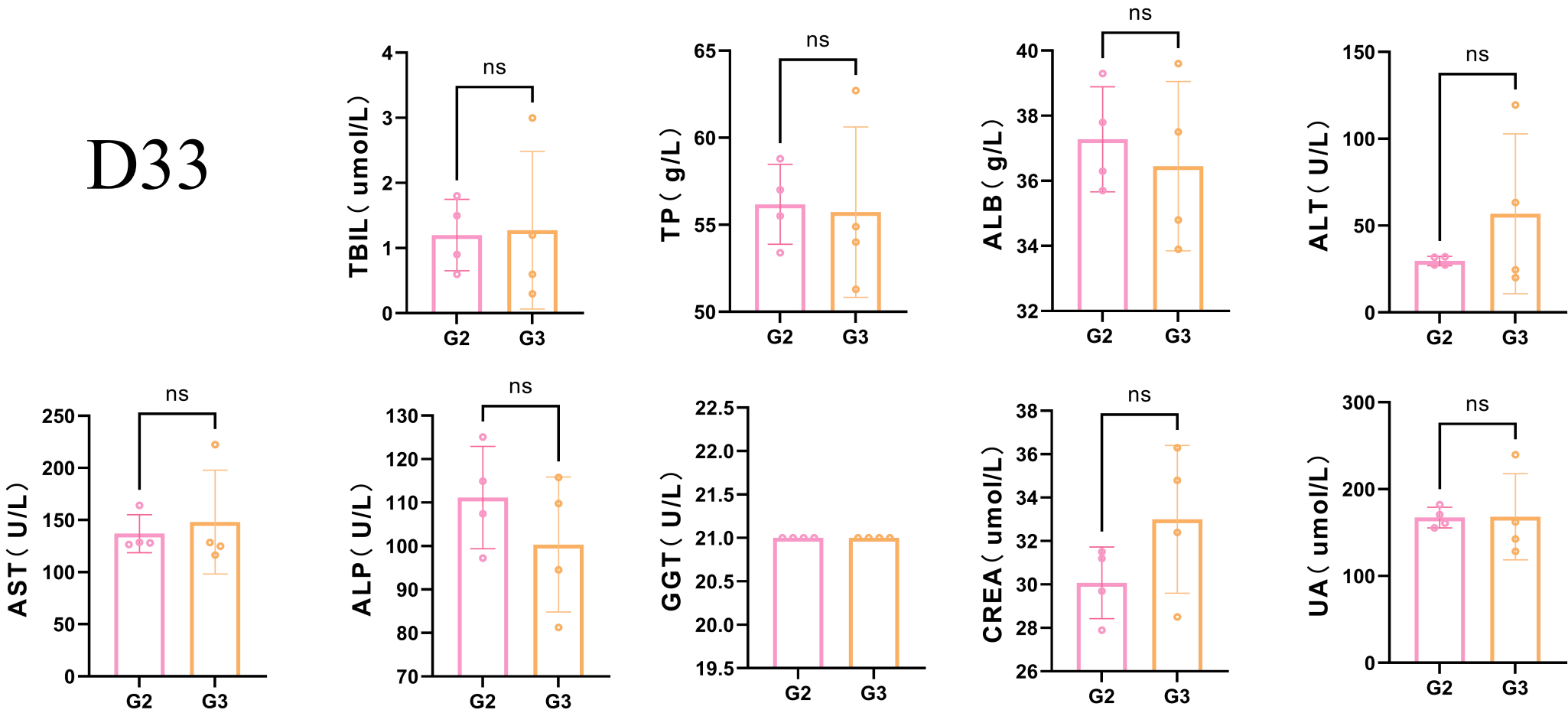

D60

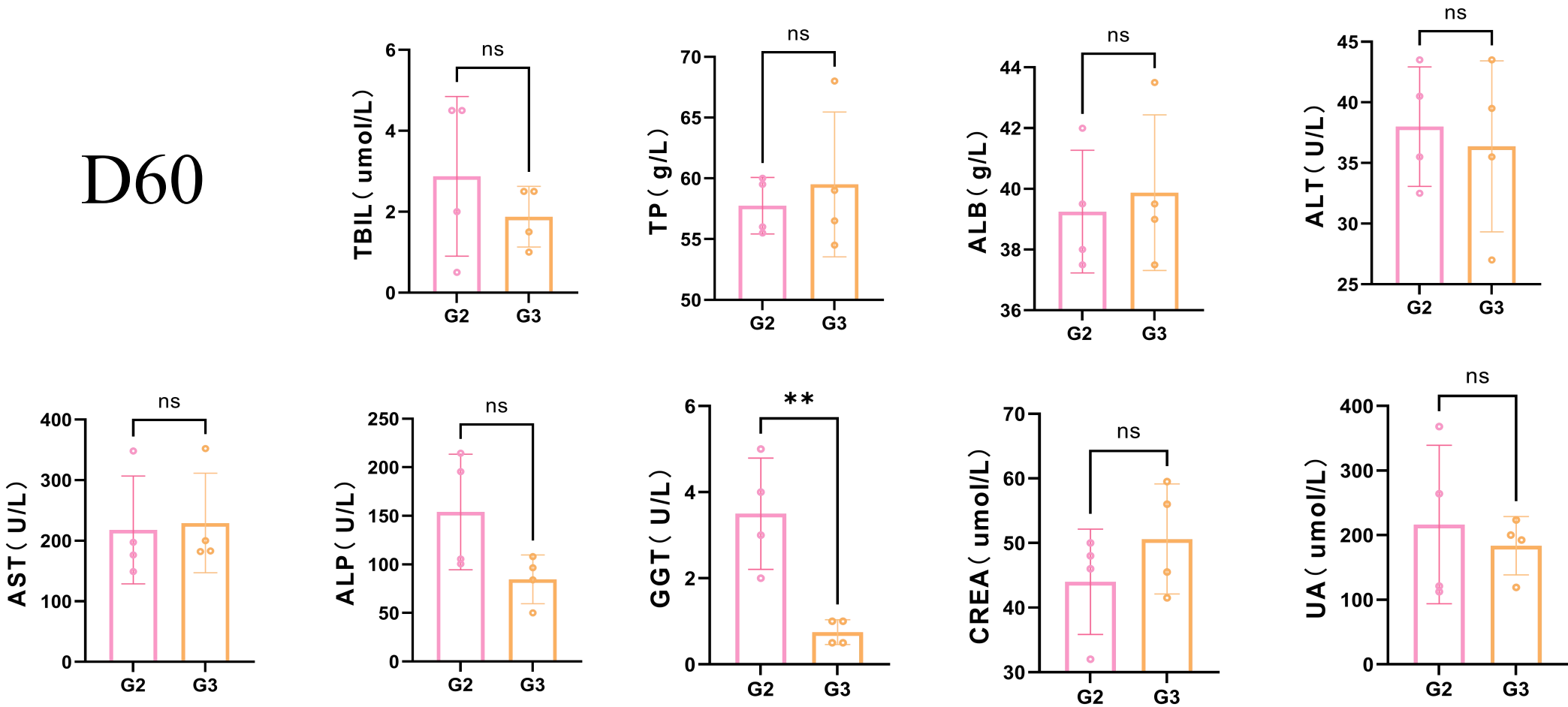

Supplement: Supplementary file 4 — Supporting Information [file CTM2-15-e70264-s001.pdf]
