# Supplementary material for: Chimeric antigen receptor T cell therapy based on stem cell‐like memory T cells enhances anti‐tumour effects in multiple myeloma
Source: Clin Transl Med. 2025 Mar 5;15(3):e70264. doi: 10.1002/ctm2.70264 (PMC11882385; doi:10.1002/ctm2.70264)
Supplement: Supplementary file 5 — Supporting Information [file CTM2-15-e70264-s004.docx]

Below is the link to the electronic supplementary material：

Supplementary Material 1：


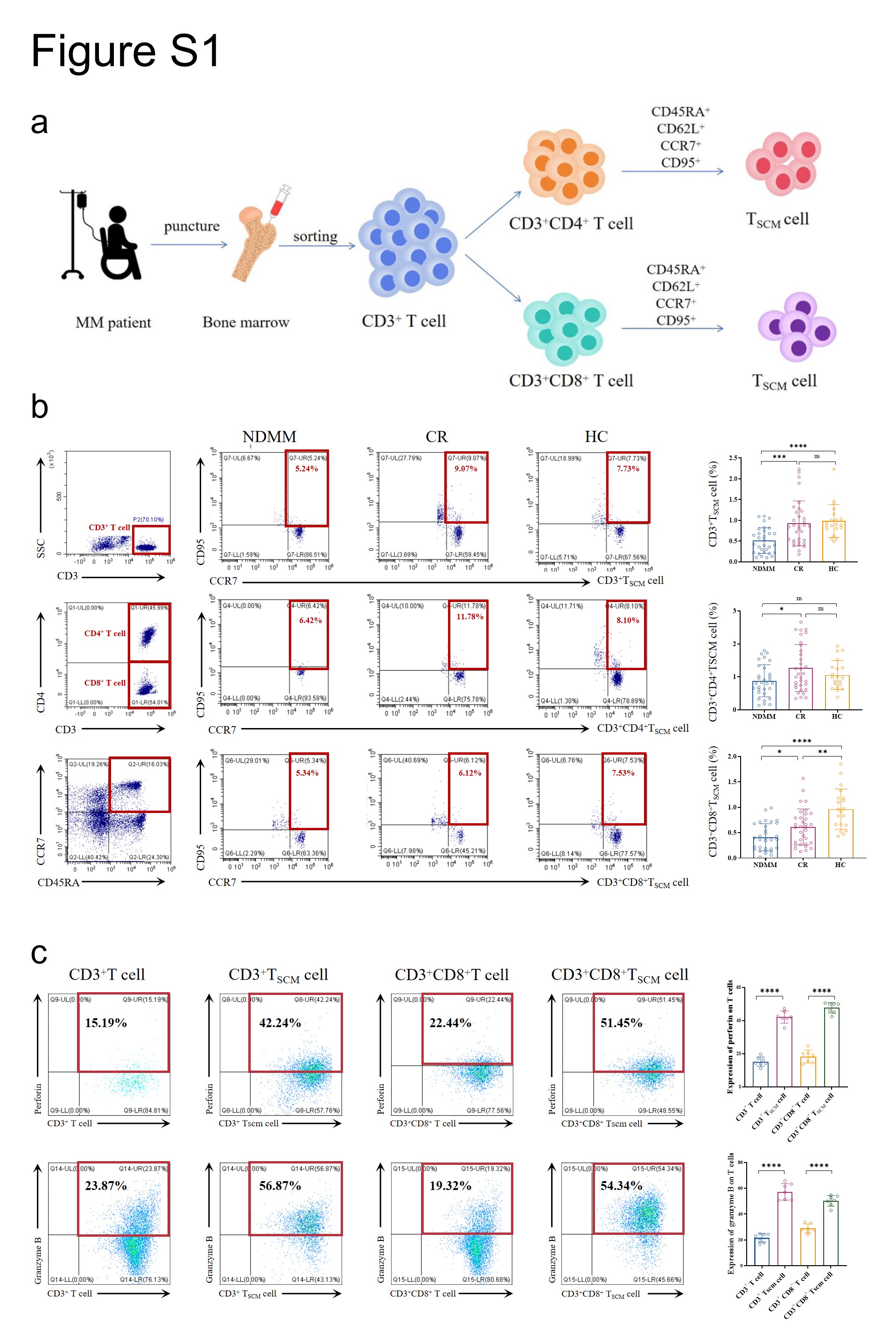


Figure S1 The number of T_SCM_ cells in MM patients is low but exert a stronger function. a.T_SCM_ cells are a subpopulation of T cells with a low degree of phenotypic differentiation. b.FCM detected the percentage of CD3^+^TSCM cell、CD3^+^CD4^+^T_SCM_ cell and CD3^+^CD8^+^ T_SCM_ cell in the bone marrow of patients in the HC, NDMM and CR groups. c.FCM detects the function of CD3^+^T cell、CD3^+^T_SCM_ cell、CD3^+^CD8^+^T cell and CD3^+^CD8^+^T_SCM_ cell in the bone marrow of patients with NDMM.

*P<0.05; **P<0.01; ***P<0.001;****P<0.0001; ns: not signifcant.

Supplementary Material 2：


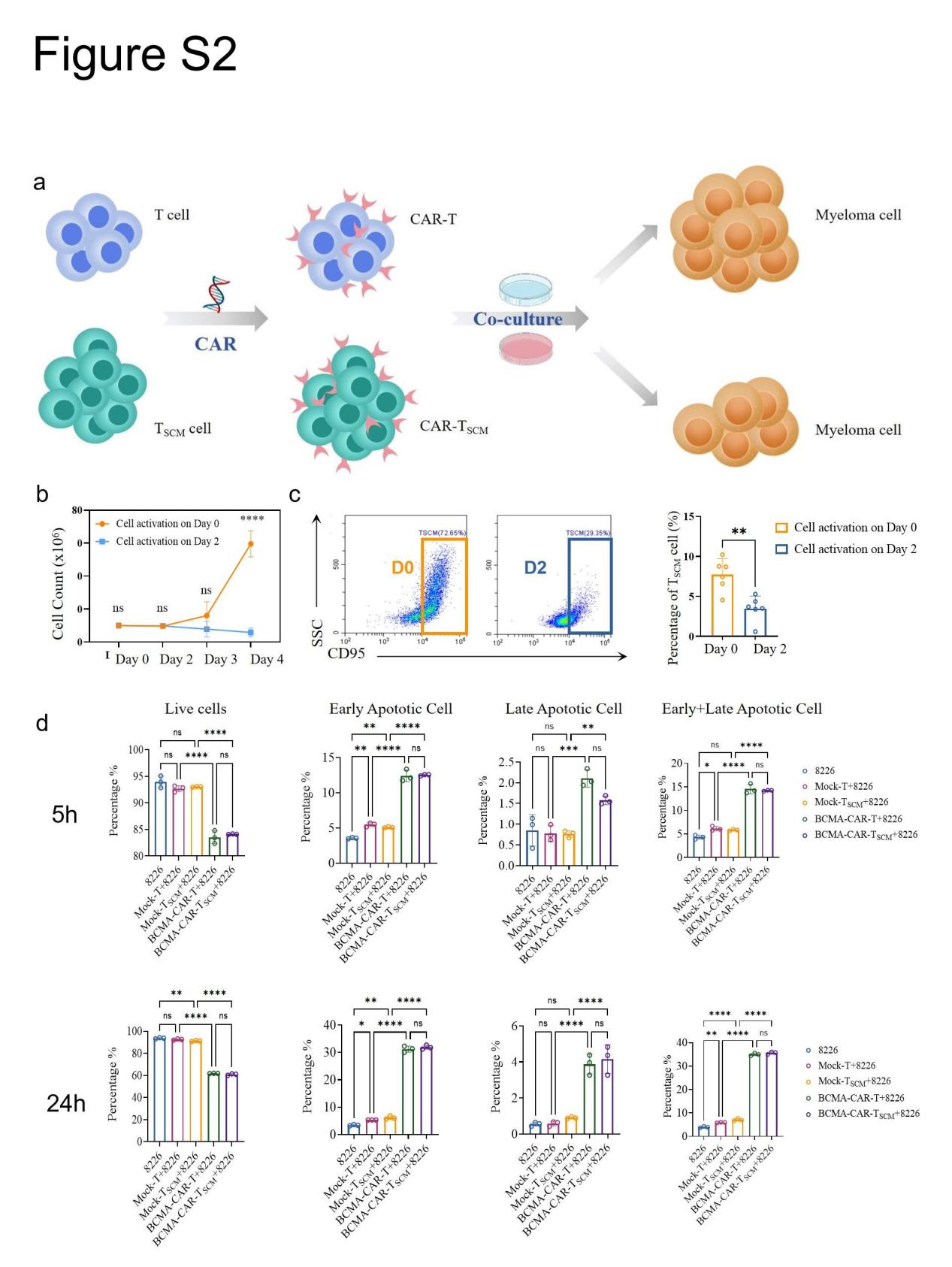


Figure S2 T_SCM_ cells activated with T cells at day 0 and transfected with CAR after expansion exerted better anti-tumour effects. a. Schematic representation of in vitro experiments to validate the anti-tumor effect of CAR-T_SCM_ cells. b. Comparison of the number of T_SCM_ cells counted microscopically after activation of T cells with T Cell TransAct™ on day 0 and day 2. c. FCM verifies the difference in the percentage of T_SCM_ cells after activation of activated T cells on day 0 and activated T cells on day 2. d. FCM verified the differences in apoptosis levels of RPMI-8226 cells after 5h and 24h in vitro co-culture with MM cell line RPMI-8226 under different conditions.

*P<0.05; **P<0.01; ***P<0.001;****P<0.0001; ns: not signifcant.

Supplementary Material 3：


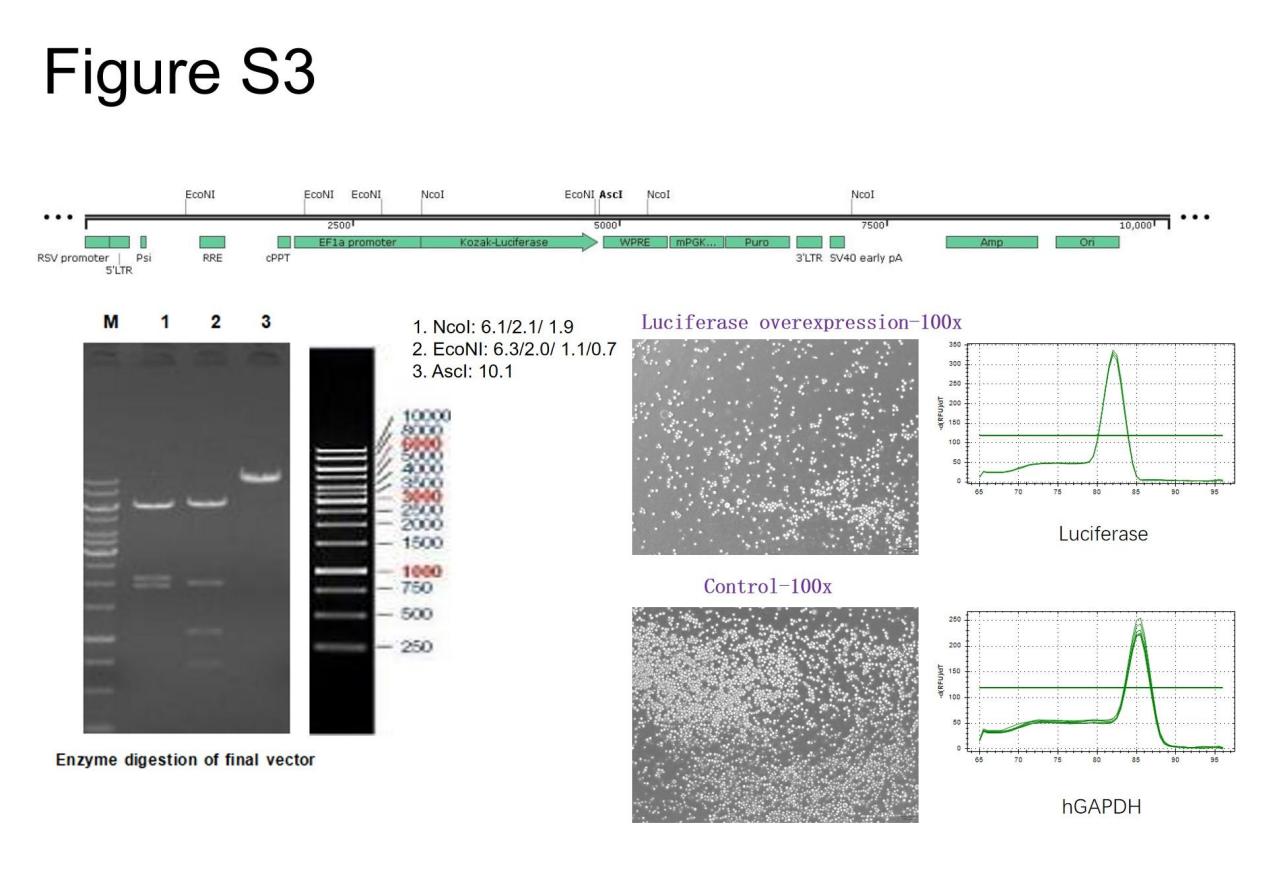


Figure S3 Construction method and validation of RPMI-8226 Luciferase Overexpression stable transcript strain

Supplementary Material 4：


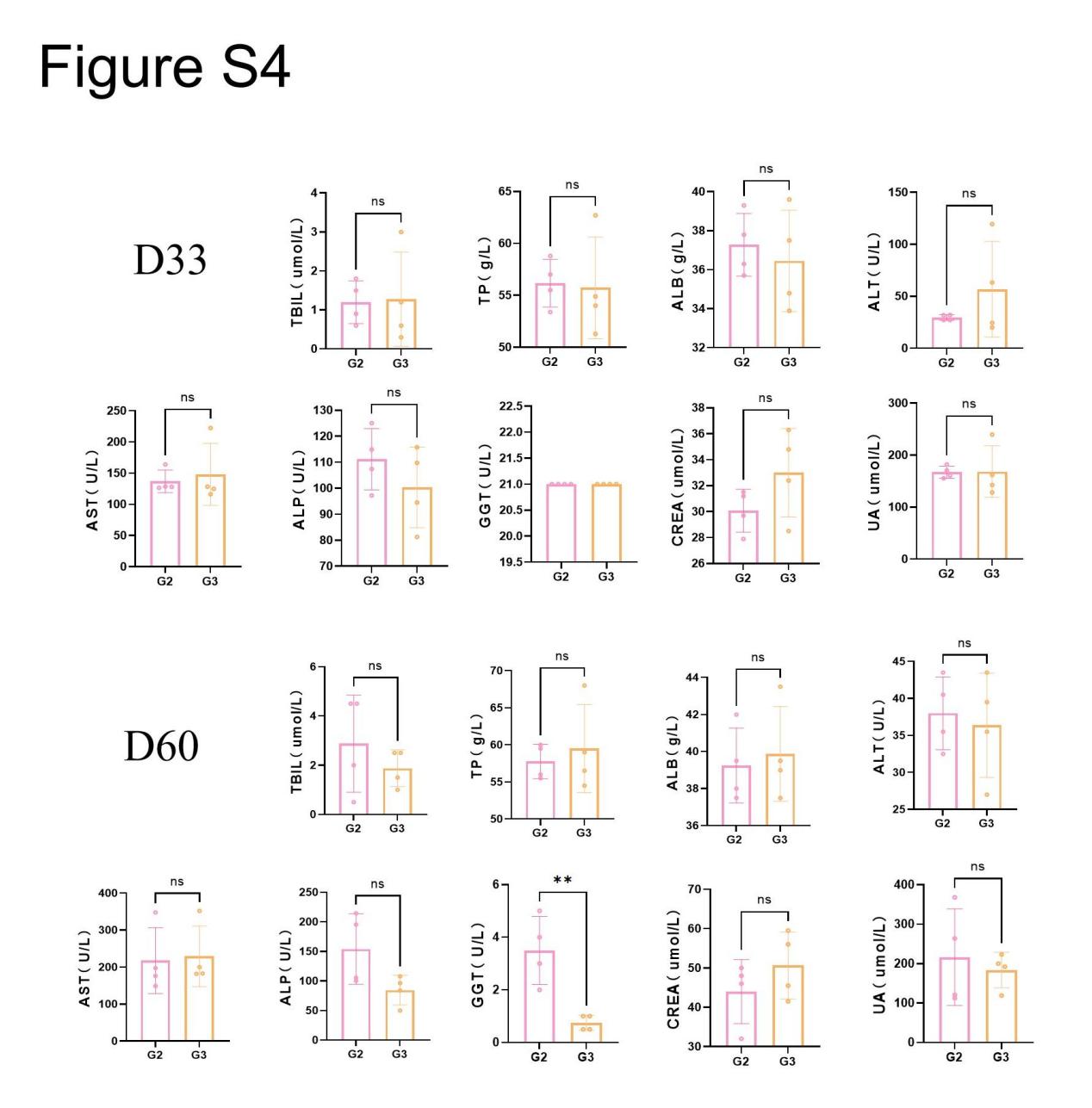


Figure S4 Comparison of liver and kidney index after blood sampling from the tail vein of mice in the G2 group (BCMA-CART treatment group) and G3 group (CAR-T_SCM_ treatment group) at D33 and D66.
